# From mapping to clipping: The role of thoracoscopic left atrial appendage clipping in refractory focal left atrial tachycardia

**DOI:** 10.1016/j.hrcr.2026.01.003

**Published:** 2026-01-14

**Authors:** Taesoon Hwang, Sandeep Panikker, Jassie Tan, Jamal Khan, Will Foster, Thomas Barker, Faizel Osman, Ven Gee Lim

**Affiliations:** 1 Department of Cardiology, University Hospitals Coventry and Warwickshire NHS Trust, Coventry, United Kingdom; 2 Institute for Cardio-Metabolic Medicine, University Hospitals Coventry and Warwickshire, Coventry, United Kingdom; 3 Warwick Medical School, University of Warwick, Coventry, United Kingdom; 4 Worcester Acute Hospitals NHS Trust, Worcester, United Kingdom

**Keywords:** Thoracoscopic clipping, Atrial tachycardia, Left atrial appendage, Drug-refractory arrhythmia, Rhythm control

**Key Teaching Points**

• Thoracoscopic left atrial appendage (LAA) clipping is a safe, effective, and minimally invasive treatment for drug- and ablation-refractory atrial tachycardia (AT) originating from the LAA, offering durable electrical isolation and eliminating the need for long-term antiarrhythmic therapy.
• Radiofrequency ablation of LAA-origin AT carries significant risks, including perforation and thromboembolism.
• This case broadens the clinical scope of LAA clipping by demonstrating its safety and efficacy in a structurally normal heart, extending its use beyond previously reported high-risk or anatomically complex scenarios.

## Introduction

Focal atrial tachycardia (AT) arising from the left atrial appendage (LAA) is rare, and its management remains challenging, especially in drug- and ablation-refractory cases. In this case report, surgical LAA clipping provided a novel therapeutic approach, achieving sinus rhythm (SR) and symptom relief.

## Case report

A 19-year-old woman presented with paroxysmal AT characterized by daily palpitations lasting up to 2 hours and associated with fatigue and exertional dyspnea, which significantly limited her functional capacity and adversely affected her quality of life.

Her other medical history included a small patent ductus arteriosus (2.6 × 2 mm) with no right heart dilatation and previous laparoscopic ablation for endometriosis.

**Figure 1:**
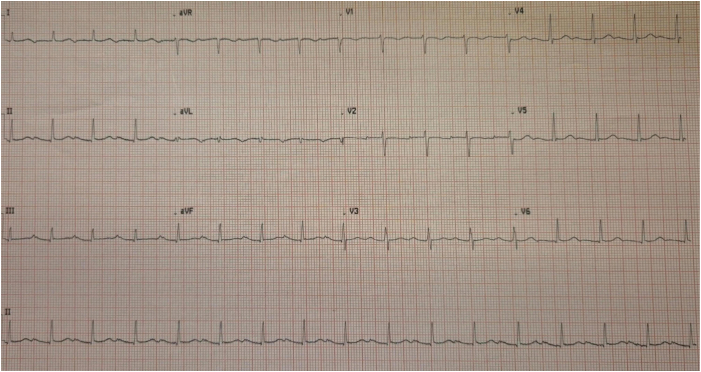
12-lead electrocardiogram demonstrating atrial tachycardia with a ventricular rate of 97 beats per minute. Positive P wave in V1, bifid P wave in lead II, negative P wave in lead I localize the focus of tachycardia to either the left atrial appendage or the left pulmonary vein as per the Kistler algorithm.

Her baseline blood tests were unremarkable, and a 12-lead electrocardiogram obtained during a clinic appointment confirmed AT with a borderline fast ventricular rate of 97 beats per minute (Figure 1). The P-wave morphology revealed a positive deflection in lead V1, a bifid P wave in lead II, and a negative P wave in lead I, localizing the arrhythmic focus to either the LAA or the left pulmonary vein as per the Kistler algorithm.^1^ Her transthoracic echocardiography demonstrated a structurally normal heart.

Despite initial pharmacologic treatment with flecainide and nebivolol, symptom control and rate management remained suboptimal. Given her young age and recurrent symptomatic episodes despite medical therapy, she underwent a cardiac electrophysiological (EP) study.

At the start of her EP study, she was in AT with eccentric atrial activation consistent with a left atrial origin and a ventricular rate of 96–100 beats per minute. However, the tachycardia spontaneously terminated to SR early in the study, and despite extensive pacing protocols and pharmacologic provocation with isoprenaline and atropine, the AT could not be reinduced. Hence, no ablation was performed during this session.

**Figure 2:**
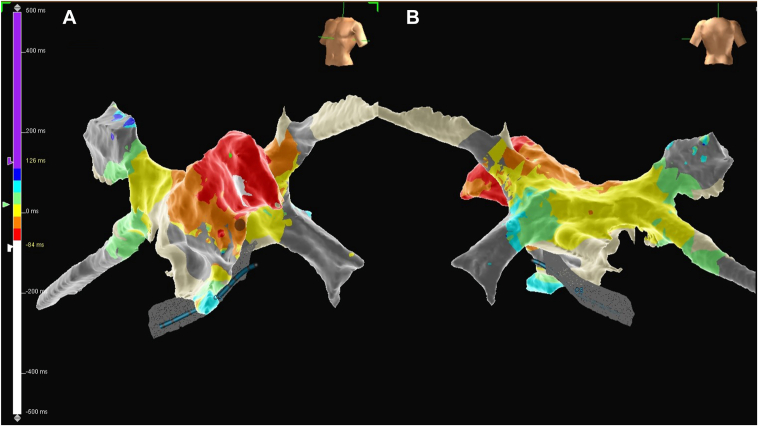
Local activation time map of the left atrium created during atrial tachycardia using the EnSite system. **A:** Anterior view. **B:** Posterior view. The earliest activation site (red) is located at the distal tip of the left atrial appendage with centrifugal propagation toward the rest of the left atrium.

**Figure 3:**
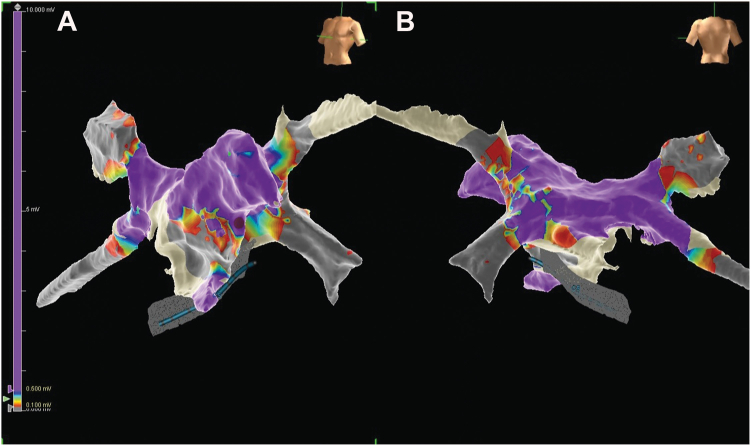
Voltage map of the left atrium using the EnSite system. **A*:*** Anterior view. **B:** Posterior view. The map demonstrates a relatively healthy left atrium.

Owing to ongoing symptomatic recurrences of AT, the patient underwent a second EP study and was in persistent AT at baseline. A 3-dimensional electroanatomic map of the left atrium was constructed using the EnSite system (Abbott), incorporating local activation time, voltage, and sparkle mapping. The local activation time map (Figure 2) localized the earliest site of activation to the distal tip of the LAA, which occurred at −70 ms compared with the coronary sinus, and this was further corroborated on a sparkle map (Supplemental Video 1). The voltage map (Figure 3) revealed a relatively healthy left atrium. Subsequently, a total of 26 radiofrequency ablations (TactiCath, Abbott) were delivered within the distal LAA at a power of 30 W over a cumulative duration of 20 minutes and 29 seconds; however, the patient remained in AT. Owing to the increased procedural risk associated with further ablation in the LAA, including the potential for perforation and thromboembolism from LAA isolation, the procedure was abandoned.

3 months later, the patient continued to experience symptomatic AT despite treatment with regular flecainide and nebivolol. Given the ongoing symptom burden, the case was reviewed at a larger multidisciplinary team meeting involving both the cardiology and cardiothoracic surgery teams. 3 treatment strategies were proposed: (1) repeat ablation with percutaneous occlusion of the LAA, (2) standalone thoracoscopic surgical LAA occlusion, and (3) continued pharmacologic treatment, which had already demonstrated limited efficacy.

In view of the anatomic challenges and procedural risks associated with the percutaneous approach, thoracoscopic LAA clipping was considered the most appropriate option, offering effective electrical exclusion of the LAA while minimizing risks of perforation and tamponade. Surgical clipping was also anticipated to offer a more complete and durable mechanical closure of the LAA compared with percutaneous occlusion devices, further mitigating thromboembolic risk. In addition to the multidisciplinary team’s recommendation, the patient’s preferences were carefully considered as she expressed a strong desire for a definitive yet minimally invasive solution. After a detailed discussion of the risks and benefits of each treatment option, the patient elected to proceed with the thoracoscopic LAA clipping.

**Figure 4:**
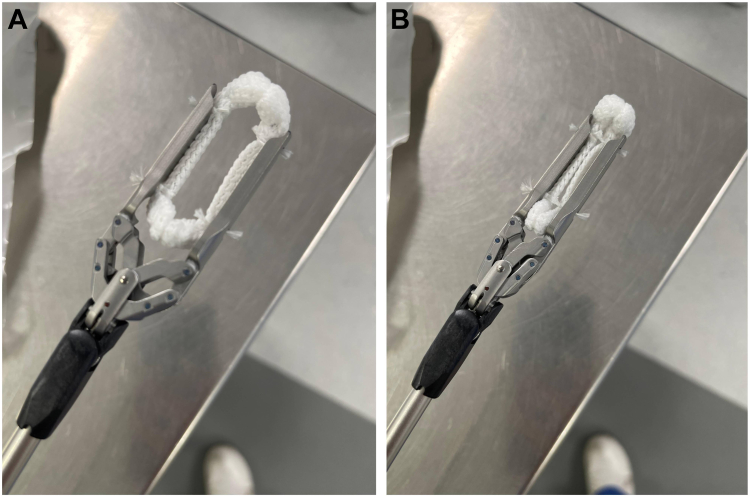
Surgical clip used in left atrial appendage occlusion. **A:** Open position. **B:** Closed position.

**Figure 5:**
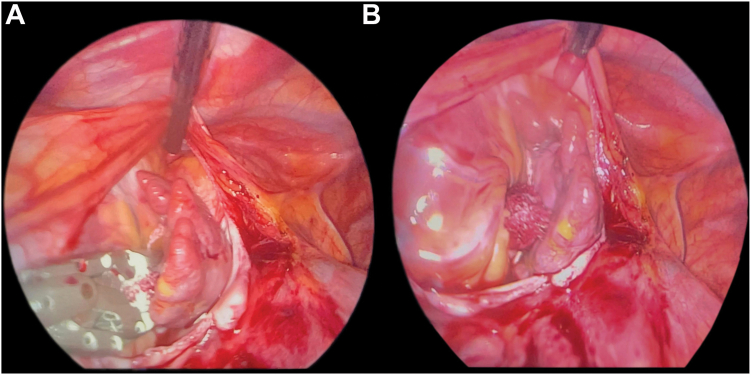
Intraoperative thoracoscopic views of left atrial appendage (LAA) clipping. **A:** Preclosure: the LAA was visualized, and the surgical clip was positioned at its base. **B:** Postclosure: the LAA is completely excluded with the clip securely in place across its base.

Surgical LAA clipping was performed under general anesthesia. A transesophageal echocardiogram confirmed the absence of thrombus within the LAA and demonstrated an appendage length of 21 mm. 3 left lateral ports were then introduced: a camera port at the fourth intercostal space and 2 working ports at the second and sixth intercostal spaces. The course of the left phrenic nerve was identified, and pericardiotomy was performed below the nerve to expose the LAA. Subsequently, a 35 mm surgical clip (AtriCure Inc) (Figure 4) was deployed across the base of the LAA (Figure 5, Supplemental Video 2), and its complete closure was confirmed on the intraoperative transesophageal echocardiogram with no remnant stump. Given that the procedure was uneventful and the postoperative chest radiograph was unremarkable, the patient was discharged 2 days after the elective surgery.

**Figure 6:**
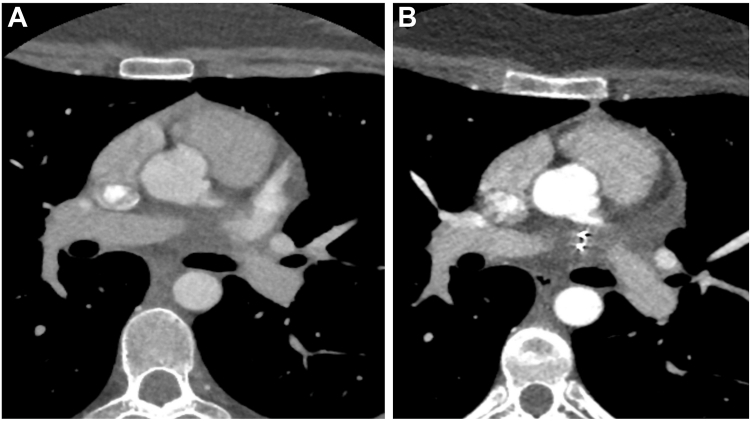
Axial computed tomography cardiac angiogram before and after left atrial appendage (LAA) closure. **A:** Preclosure: the LAA is well opacified with contrast, indicating normal blood flow. **B:** Postclosure: a radiopaque clip is visible at the LAA ostium with no contrast filling, consistent with successful LAA exclusion.

**Figure 7:**
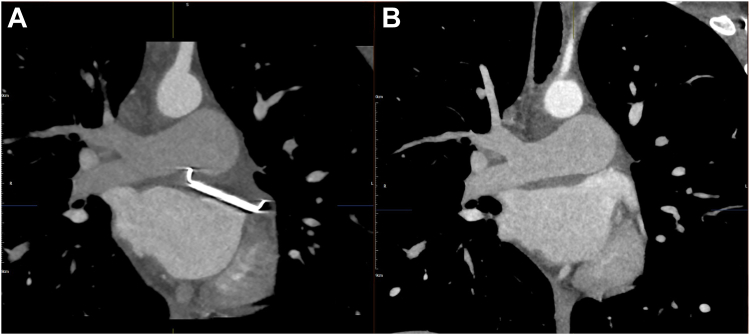
Oblique coronal computed tomography cardiac angiogram demonstrating left atrial appendage (LAA) closure. **A*:*** Postclosure: a radiopaque surgical clip is seen at the LAA ostium with complete absence of contrast opacification, confirming successful exclusion. **B** Preclosure: the LAA is fully opacified with contrast, demonstrating patency and communication with the left atrium.

At the 3-month follow-up, the patient remained asymptomatic and reported a significant improvement in quality of life. Electrocardiography confirmed sustained SR, and a cardiac computed tomography angiogram further demonstrated successful occlusion of the LAA (Figures 6 and 7). Given her clinical stability, flecainide was discontinued, and nebivolol was gradually tapered. At a further review 19 months after surgical LAA occlusion, she continued to be symptom-free and maintained SR, allowing her to fully resume normal daily activities without limitation from palpitations. She expressed high satisfaction with the outcome, given that the procedure aligned with her preferences for definitive management of AT.

## Discussion

This case illustrates the successful management of refractory focal AT through a structured, stepwise approach. Thoracoscopic clipping of the LAA definitively relieved her symptoms and cured her arrhythmia while removing the need for long-term antiarrhythmic therapy. This is particularly important given her young age, given that it avoids the cumulative medication side effects and eliminates the complexities of antiarrhythmic selection during potential future pregnancies.

Although LAA clipping is primarily used for thromboembolic prophylaxis in patients with atrial fibrillation (AF) with contraindications to anticoagulation, several recent case reports have demonstrated its novel application in achieving durable electrical isolation for focal AT arising from the LAA (Table 1).^2^, ^3^, ^4^, ^5^ Given that the evidence to date is limited to individual case reports, it is premature to conclude consistent achievement of electrical isolation and long-term rhythm control with surgical LAA occlusion. However, these reports have shown encouraging results with successful termination of AT and sustained SR at follow-up intervals ranging from 6 to 12 months. Our case contributes to the emerging evidence by demonstrating SR maintenance at 19 months after surgical LAA occlusion, a longer follow-up than previously reported, and thereby supporting its durability.

**Table 1:** Summary of published case reports describing LAA occlusion for the management of focal atrial tachycardia arising from the LAA

Author (y)	Patient characteristics	Underlying cardiac substrate/structural abnormality	Type of arrhythmia	LAA intervention	Outcome	Follow-up duration
Benussi et al,^2^ 2011	15-y-old boy with incessant, drug-refractory AT; previously treated with metoprolol, verapamil, and flecainide	Structurally normal heart; normal LV size and function; normal atrial volumes	Focal AT originating from the distal LAA	Thoracoscopic LAA clipping with intraoperative confirmation of electrical isolation	SR	6 mo
Combes et al,^3^ 2018	55-y-old woman with incessant AT; previously failed 2 ablation procedures for AT	Giant LAA with otherwise normal left atrium; previous AF ablation	Focal AT originating from the LAA	Thoracoscopic clipping with intraoperative confirmation of electrical isolation	SR	6 mo
Queirós et al,^5^ 2021	35-y-old woman with incessant AT and a history of pulmonary sarcoidosis; refractory to beta-blocker therapy	Mildly reduced LVEF (≈39%) owing to tachycardia-induced cardiomyopathy	Focal AT originating from the distal LAA	Thoracoscopic LAA clipping	SR	1 y
Melman et al,^4^ 2024	46-y-old man with incessant AT and admitted with cardiogenic shock; failed cardioversion, amiodarone, ivabradine, and ablation	Tachycardia-mediated cardiomyopathy with severely reduced EF (≈10%)	Focal AT originating from the distal LAA	Thoracoscopic LAA clipping	SR, EF 45%	1 mo

AF = atrial fibrillation; AT = atrial tachycardia; EF = ejection fraction; LAA = left atrial appendage; LV = left ventricle; LVEF = left ventricular ejection fraction; SR = sinus rhythm.

In addition to longer follow-up, the novelty of our case report lies in its broader clinical applicability. Previous reports of LAA clipping for LAA-mediated AT have predominantly involved patients with underlying structural or functional cardiac abnormalities and incessant AT. For example, Combes et al^3^ described a case of focal AT arising from a giant LAA in a patient with a history of AF; Queirós et al^5^ reported incessant AT in a patient with mildly reduced left ventricular ejection fraction (≈39%) owing to tachycardia-induced cardiomyopathy; and Melman et al^4^ presented a case complicated by cardiogenic shock and severely impaired left ventricular ejection fraction (≈10%). In contrast, to the best of our knowledge, our case is the first reported instance of thoracoscopic LAA clipping performed for paroxysmal LAA-mediated AT in a patient with a structurally normal heart and clinically stable condition.

This case further highlights the anatomic and procedural challenges of managing AT originating from the LAA. Although radiofrequency ablation of AT generally demonstrates high success rates (70%–90%), these figures do not fully reflect the complexity or risks associated with LAA-mediated AT, which is relatively rare and accounts for only approximately 2% of focal AT cases.^6^^,^^7^ The thin appendage wall increases the risk of perforation and cardiac tamponade during ablation, whereas electrical isolation increases thromboembolic risks.^8^^,^^9^ Moreover, variable tissue thickness and complex trabeculations within the appendage render catheter contact technically challenging, thereby predisposing to ineffective lesion formation. In this case, the earliest activation at the distal LAA (−70 ms before the coronary sinus) strongly supported proximity to the focus; however, failure to terminate AT despite extensive ablation suggested a deeper or epicardial source inaccessible to endocardial ablation. Given the increased perforation risk, particularly after multiple endocardial lesions, epicardial ablation was deemed unsafe in this patient.

A key limitation of this report is the absence of an intraoperative EP study, because our institution does not routinely incorporate intraoperative mapping, given that the procedure is mostly performed for stroke prevention in AF. Consequently, we were unable to directly confirm electrical isolation of the LAA or the precise mechanism by which AT suppression occurred. Nonetheless, the preprocedural findings allow us to infer the most plausible explanation.

To determine the likely mechanism of arrhythmia suppression, it is necessary to first understand the mechanism of AT itself. Sparkle mapping demonstrated centrifugal spread from a discrete focal site at the LAA, consistent with either abnormal automaticity or triggered activity rather than reentry, especially given the preserved atrial voltages. Moreover, neither isoprenaline infusion nor pacing, both of which typically facilitate triggered activity by promoting Ca^2+^ overload, was able to reproduce AT during the EP study.^10^^,^^11^ This supported abnormal automaticity as the predominant mechanism rather than triggered activity, and the limited efficacy of antiarrhythmic therapy further supported this. Flecainide, which suppresses sodium current to prolong atrial refractoriness, and nebivolol, which has been shown to reduce spontaneous Ca^2+^ release and delayed afterdepolarization, both provided minimal clinical benefit.^12^^,^^13^ Such poor response makes reentry and triggered activity less likely, reinforcing abnormal automaticity as the most plausible underlying mechanism.

Once a focal LAA source was identified, clip exclusion provided a logical therapeutic strategy. By compressing the appendage base, the clip induces immediate conduction block and, over time, leads to tissue necrosis and fibrosis that reinforces durable electrical isolation of the arrhythmogenic focus. This is consistent with the intraoperative studies by Benussi et al^2^ and Combes et al,^3^ who demonstrated an immediate electrical isolation after clip deployment, confirmed by pacing maneuvers showing conduction block between the LAA and the left atrium. Hence, this mechanism most plausibly explains the prompt restoration and long-term maintenance of SR observed in our patient.

## Conclusion

Overall, this case demonstrates the safety and efficacy of thoracoscopic LAA clipping in the treatment of drug- and ablation-refractory focal AT arising from the LAA. It further underscores its applicability in a clinically stable patient with preserved cardiac function and paroxysmal AT, offering durable rhythm control and improved quality of life.

## Disclosures

The authors have no conflicts of interest to disclose.

## Footnotes

Appendix Supplementary data associated with this article can be found in the online version at https://doi.org/10.1016/j.hrcr.2026.01.003.
