# Supplementary material for: From mapping to clipping: The role of thoracoscopic left atrial appendage clipping in refractory focal left atrial tachycardia
Source: HeartRhythm Case Rep. 2026 Jan 14;12(4):385–91. doi: 10.1016/j.hrcr.2026.01.003 (PMC13100621; doi:10.1016/j.hrcr.2026.01.003)
Supplement: Supplementary Material [file mmc3.docx]

Video 1. Sparkle map of the left atrium during atrial tachycardia using the EnSite system. (A) anterior view (B) posterior view. The map demonstrates focal activation originating from the distal tip of the left atrial appendage and centrifugal propagation toward the remainder of the left atrial body.

Video 2. Intraoperative thoracoscopic view of left atrial appendage (LAA) clipping. This demonstrates the positioning of the surgical clip at the base of the LAA and its subsequent deployment.
